# Feasibility of modifying the washout water weir on dyna sand filters performance

**DOI:** 10.1038/s41598-026-41574-4

**Published:** 2026-03-24

**Authors:** Esraa Mahmoud Ahmed El Taher, Mohamed El Hosseiny El Nadi, Mohamed Nouh Ahmed Meshref, Amira Mohamed Nagy

**Affiliations:** Faculty of Engineering, ASU, Cairo, Egypt

**Keywords:** Dyna-sand filter, Removal efficiency, Operational stability, Water production optimization, Washout weir modification, Engineering, Environmental sciences, Hydrology

## Abstract

Among the available technologies, the dyna-sand filter has gained attention due to its continuous filtration and sand washing mechanism, which provides an advantage in maintaining stable operation. Nevertheless, its performance under variable heights of washout weir with constant solid loads and the optimization of its washing system remain areas that require further study. This study investigates the use of the dyna-sand filter in water treatment facilities, focusing on the alteration of the washout weir to improve efficiency and reduce washout water amount. The study evaluates the filter’s performance under fixed influent total suspended solids (TSS) concentration and constant filtration rate (ROF), aiming to demonstrate the advantages of this alteration in enhancing removal efficiency, operational stability, and water saving through a laboratory-scale pilot. The average washout discharge fell from 0.788 to 0.486 L/min, a 38.3% reduction in washout water amount. As a result, filtered water production rose by nearly 2.2%, indicating more efficient hydraulic operation. These findings confirm that raising the washout weir by 4 cm improved filtration and reduced washout water loss. This, in turn, enhanced system productivity and the quality of washwater produced, assuming a consistent flow rate and solid load.

## Introduction

Water treatment efficiency is one of the most critical challenges facing modern environmental engineering, especially with the continuous increase in water demand and the deterioration of surface water quality. Conventional filtration systems, though widely applied, often suffer from limited adaptability under fluctuating solid loads and variable hydraulic conditions. This has led researchers to create more advanced and sustainable filtration technologies that can maintain high performance with minimal operational complexity^1^.

Efficient solid–liquid separation is a fundamental step in both water and wastewater treatment processes, as it directly influences effluent quality and the overall performance of treatment plants. Conventional filtration systems, such as rapid sand filters, often face limitations related to head loss, frequent backwashing, and restricted flexibility under varying solids loading. To overcome these challenges, continuous filtration systems such as the dyna-sand filter have been introduced, offering steady operation with automatic sand cleaning and reduced downtime^2^.

This lack of focused investigation represents a critical research gap, given the direct role of washout hydraulics in governing solids transport and filter bed behavior.

In this study, a laboratory-scale filtration unit was designed and constructed to simulate the operation of a dyna-sand system to evaluate the impact of washout weir height on filtration performance under controlled conditions^3^.

The experimental program was conducted at a constant filtration rate of 300 m³/m²/d, while influent total suspended solids (TSS) concentrations and washout water head were systematically varied to assess their effects on removal efficiency, effluent quality, and washout water consumption^4^.

The novelty of this study lies in providing a data-driven optimization of washout weir height using combined experimental, graphical, and empirical analyses. By explicitly linking washout hydraulics to filtration efficiency and hydraulic recovery, the study offers practical insights that support improved operational control of continuous sand filtration systems in both water and wastewater treatment applications^5^.

## Literature review

The dyna-sand filter is a continuous, up-flow sand filtration system designed for efficient solid–liquid separation with minimal interruption during operation. Its distinctive feature lies in the continuous sand washing mechanism, which allows the filter to operate without shutdown or backwash cycles^6^.

This design provides high operational stability, consistent effluent quality, and reduced water loss compared to conventional rapid sand filters. Owing to these advantages, the dyna-sand system has been widely applied in both water and wastewater treatment processes, as well as in industrial and agricultural applications^7^.

In this study, the dyna-sand filter was modified to operate as a dual media filter, incorporating a combination of sand and activated carbon as the filtration media. The use of dual media aims to enhance removal efficiency by combining the mechanical filtration capacity of sand with the adsorptive properties of activated carbon^8^.

This configuration is expected to improve turbidity and TSS removal, as well as reduce organic and residual contaminants in the treated effluent. The dual media design provides a promising approach for optimizing continuous filtration performance and expanding the applicability of the DSF in advanced water treatment operations^9^.

Previous studies have highlighted the importance of filtration rate in determining overall filter efficiency. Harris (1970) reported that high-rate filtration can achieve effective solid removal when the operational parameters are well-controlled, emphasizing the balance between flow rate and media performance. This finding supports the current study’s focus on optimizing the dyna-sand filter under different hydraulic conditions to maintain high removal efficiency and operational stability^10^.

In 1993, a pilot study at the Kralingen Drinking Water Production Plant in Rotterdam, Netherlands, showed that the Dyna-Sand continuous-backwash filtration system could be used successfully for treating drinking water. The study confirmed the system’s ability to maintain stable effluent quality under varying solids loads and highlighted the critical role of controlling sand circulation rates to ensure consistent performance^11^.

In a trial study at the El Fostat Water Treatment Plant on the River Nile in South Cairo, Mostafa (2007) found that the dyna-sand system beat the traditional Pulsator–rapid sand filter in eliminating bacteria, aluminum, and algae, and it was able to remove 99% of the algae. A total cost reduction of 51.7% was achieved by the system, which also required a reduced dosage of alum and offered notable savings in footprint, construction, and operating expenses^12^.

Later, in 2008, as part of Enhanced nutrient removal (ENR) experiments, a dyna-sand pilot unit was tested in Fitzgerald Creek in the United States. The system demonstrated exceptional effluent quality in terms of suspended particles, nutrients, and turbidity when combined with Lamella settling, demonstrating its appropriateness for sophisticated wastewater treatment applications^13^.

A subsequent technical investigation in 2009 examined the hydraulics and sand-bed dynamics of continuously backwashed upflow filters, concluding that proper hydraulic regulation and media circulation are essential to maintaining high removal efficiency and operational stability. The study provided valuable design guidance for full-scale applications^14^.

The EcoWash retrofit for dyna-sand filters was first used in Laurel, Delaware, in 2011 after being developed by Parkson Corporation in 2010. The system demonstrated the potential of (DSF) technology as an environmentally friendly and energy-efficient filtration solution by successfully lowering reject water generation and power usage while preserving effluent quality^15^.

According to Hamdy (2011), a pilot dyna-sand unit enhanced sludge stability at lower influent loads and efficiently removed suspended solids up to 200 mg/L. The study verified the filter’s appropriateness under varying operating settings by recording removal efficiencies of 80–90% for retention durations of 0.175–0.7 h and surface loading rates of 69–274 m³/m²/d^16^.

To increase capacity while reducing land expansion, Eltaher (2020) looked into the use of dyna-sand technology to improve existing rapid sand filters. The study demonstrated effective performance at high filtration rates (200–500 m³/m²/d) and varying solids loading (50–250 mg/L), underscoring its suitability for wastewater treatment, post-sedimentation, and direct filtration. The method was also found to save construction costs by up to 90% and improve plant capacity by two to three times^17^.

Aboelkair (2021) further evaluated a pilot Dyna-sand unit under different solids loads, achieving 72% removal at high TSS (150 mg/L) and 89% at lower TSS (50 mg/L). The lower efficiency compared with conventional water supply filtration was attributed to the use of synthetic water with uniform particle size and unusually high solids loads, leading to the recommendation for further system development to enhance performance under high loading and filtration rates^18^.

Although the application differs, the study by Yussupov et al. on the decolmatation of wells highlights the fundamental role of hydraulic conditions in controlling solids mobilization and transport within porous media. By modifying the flow regime, accumulated fine particles were effectively mobilized, leading to improved permeability. This concept is directly relevant to the present study, where adjustments in washout hydraulics specifically the water layer height above the weir govern solids transport and filter bed behavior in Dyna-Sand filtration systems^19^.

Based on international applications, this study assesses the suitability of the dyna-sand filtration system for local use. The system will employ a dual-media filter consisting of sand and activated carbon. This configuration is intended to enhance the removal of fine suspended solids and improve overall filtration efficiency, particularly under varying hydraulic conditions and backwash head levels.

### Materials and methods

**Fig. 1:**
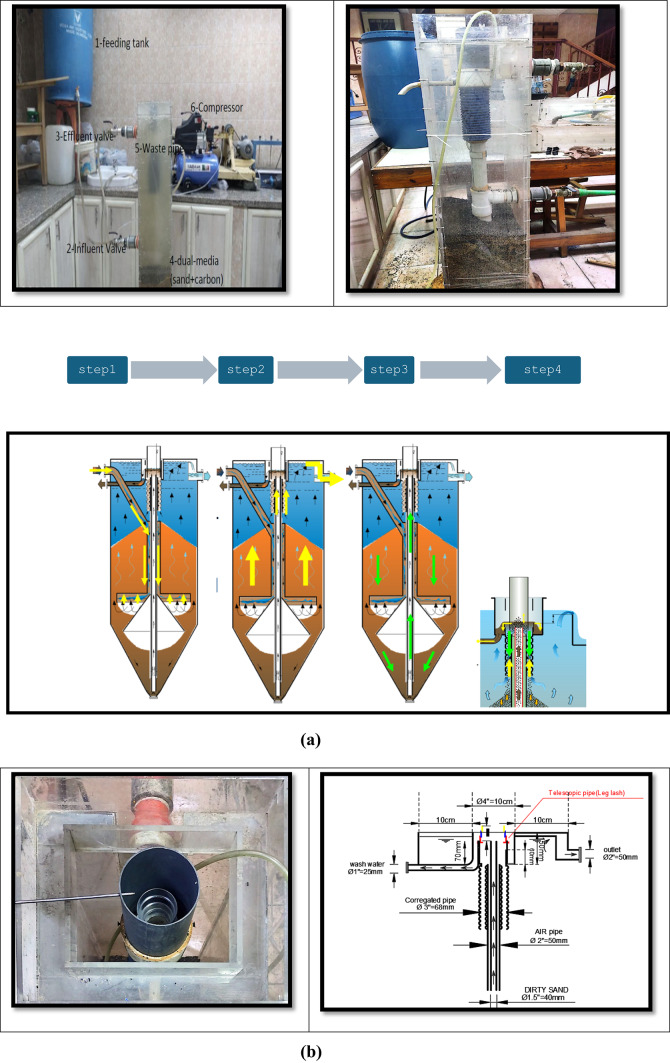
Study pilot

The study pilot was held in the Sanitary Engineering Laboratory at the Faculty of Engineering, Ain Shams University. The pilot was originally constructed during a previous research project for the dyna-sand filter unit. Figure 1 shows the pilot used inside the laboratory.

**Fig. 2:**
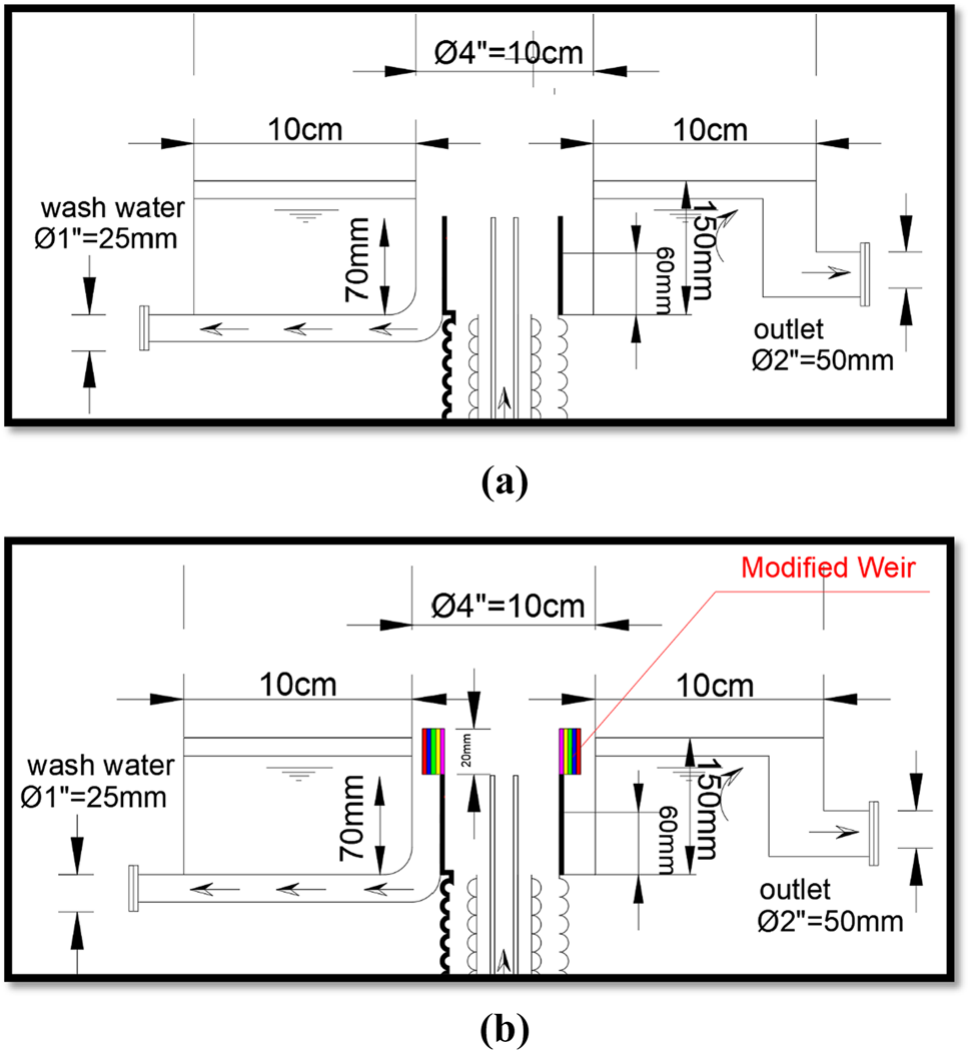
(**a**) Dyna-sand process. (**b**) After modified part in dyna-sand

The pilot unit, which simulated the dyna-sand filter, was modified by adding a movable edge to the outlet weir, allowing adjustment of the washout weir height. Figure 2a illustrates the system before modification, while Fig. 2b shows the configuration after modification. This adjustment aimed to decrease the washout water amount and increase the filter productivity as the target of this study, without affecting the filter productivity efficiency.

The primary objective of this study is to investigate the applicability of modifying the washout weir height of a dyna-sand filter and to assess its effect on reducing washout water losses and improving hydraulic recovery. Secondary objectives include evaluating the impact of washout weir modification on filtration efficiency and effluent water quality, particularly in terms of TSS removal performance. This distinction ensures that water savings and hydraulic performance remain the core focus of the study, while effluent quality improvement is considered a supporting performance indicator.

Sand media properties: The filter used sand with particle sizes ranging from 0.5 to 2 mm (D10 = 0.5 mm, D60 = 2 mm), with a uniformity coefficient (Cu) of 1.76 and a coefficient of curvature (Cc) of 1.48, classifying it as well-graded.

Filter media composition: Two sand-to-anthracite carbon ratios were tested, with results showing that increasing the carbon content to a 1:4 ratio significantly enhanced turbidity and TSS removal compared to sand-dominant configurations.

After ensuring the pilot operation stability, before the modification. Two subsequent experimental runs were then performed under a constant solid load in raw water (TSS = 250 ppm) and a fixed filtration rate of 300 m³/m²/d. The filtration unit used a dual-media bed of sand and activated carbon, providing both mechanical filtration and adsorption.

**Fig. 3:**
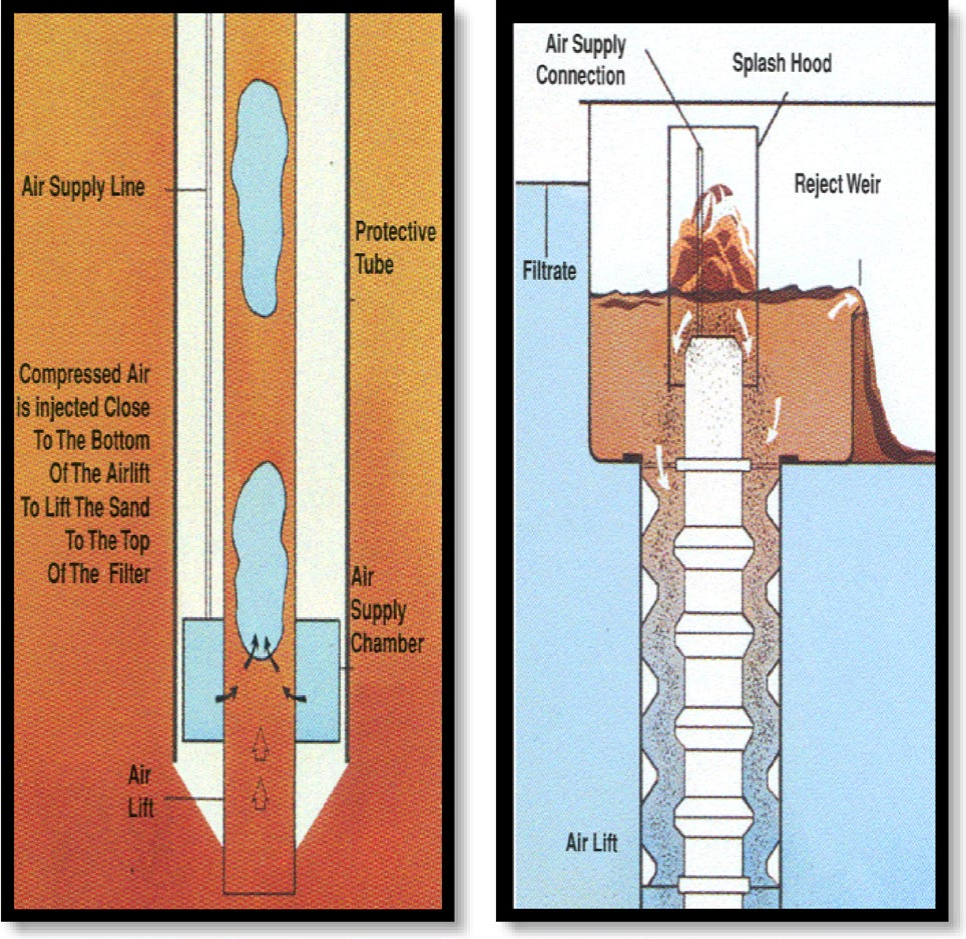
Top and bottom airlift

**Fig. 4:**
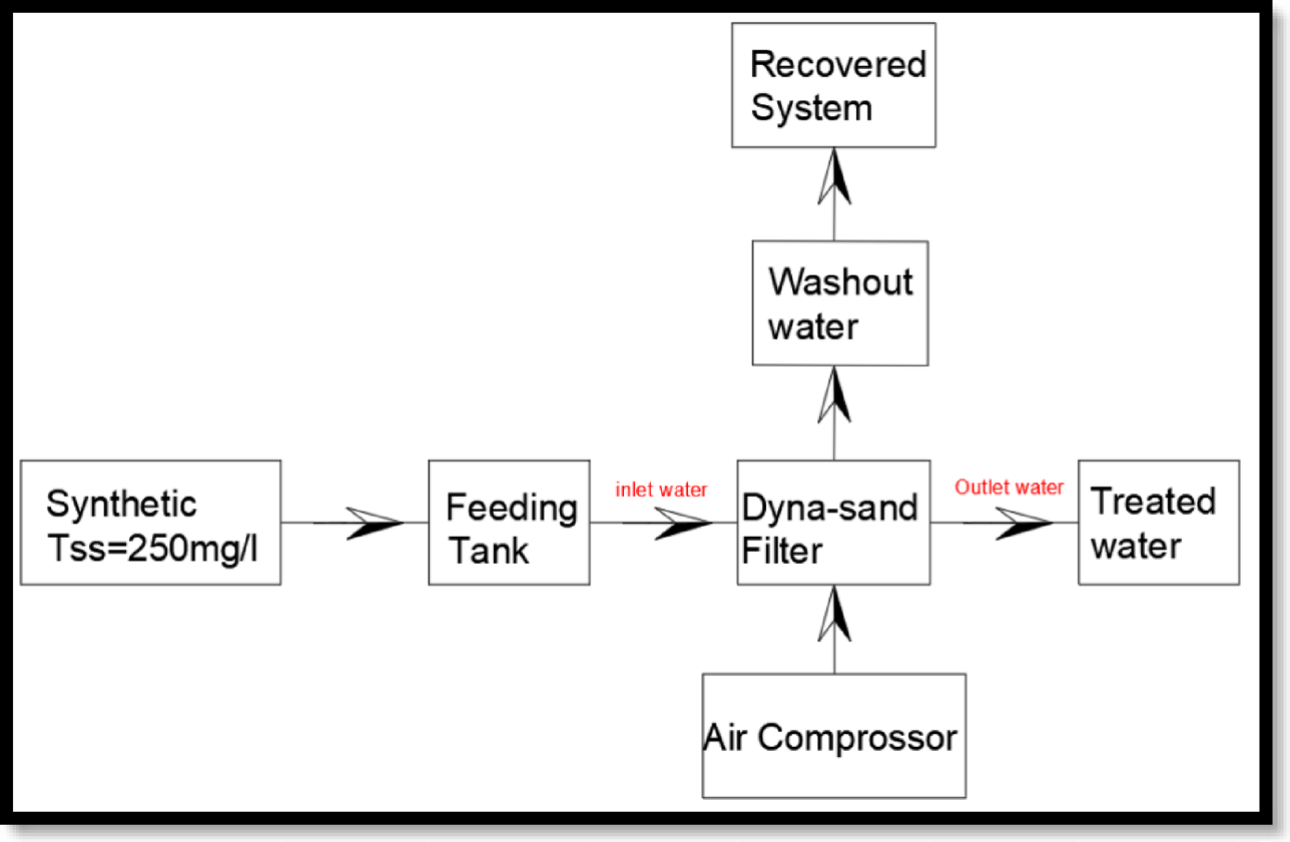
Recovered water flow chart

The experimental setup and operational performance are presented in Figs. 1B, 3 and 4, illustrates the dyna-sand process:

1. Water enters through the top of the unit and is piped through a central column down to the distribution arms, which radiate out the water evenly over the entire bed surface area.
2. Water flows up through the sand bed in which particles are trapped. After the water has been through the sand, it either flows out through the filtrate weir or up through the sand washer.
3. The action of the air lift pump pulls sand down through the filter, then up through the pump to the sand washer.
4. The sand then falls through the washer and is cleaned by the counter-current treated water. The dirty washout water then falls over a washout water weir. The difference in height.

The study comprised two runs: one without modification (R1) and another with modification (R2). Three samples were collected daily, and the mean values of the measurements were used for analysis.

Water quality measurements and lab tests followed the methods in “Standard Methods for the Examination of Water and Wastewater,” published by the American Public Health Association (APHA), American Water Works Association (AWWA), and Water Environment Federation (WEF), latest edition (APHA, 2017).

Turbidity was measured using a nephelometric method, with results in NTU. Total Suspended Solids (TSS) were measured gravimetrically after filtering through a glass fiber filter and drying at 103–105 °C. A calibrated digital pH meter was used to measure pH, and a standard laboratory thermometer was used for temperature. Flow rate (Q) was measured volumetrically using a graduated collection tank and stopwatch method to accurately characterize the filtration process^20^.

## Results and discussions

The experimental work was conducted using a laboratory-scale pilot unit; therefore, potential scale-up effects on full-scale performance should be considered. The pilot unit was designed and constructed in accordance with the manufacturer’s dyna-sand filter catalog specifications, ensuring consistency in the filtration mechanism, sand circulation, and washout hydraulics.

All experimental results were obtained from the pilot-scale study. For each day, three measurements were taken, and the daily average was calculated.

Table 1: The overall mean ± SEM for R1 was 10.42 ± 0.01 mg/L, while for R2 it was 8.80 ± 0.014 mg/L. An independent *t* test confirmed that the difference between R1 and R2 was highly significant (t = 92.90, p < 0.001).

**Table 1:** Runs (*t* test).

Run	Q_red (L/min)	Turbidity (NTU)	TSS Output (mg/L)	TSS Removal (%)	Temp (°C)	pH
R1	18.812 ± 0.010	0.76 ± 0.003	10.42 ± 0.010	95.83	24.8 ± 0.12	7.7 ± 0.06
R2	19.162 ± 0.014	0.76 ± 0.004	8.80 ± 0.014	96.47	25.8 ± 0.12	7.15 ± 0.08

Although full-scale systems may exhibit differences in hydraulic distribution, media bed behavior, and flow uniformity, the fundamental filtration and sand washing mechanisms remain unchanged. Accordingly, the observed trends—particularly the effect of washout weir height on washout water consumption, hydraulic recovery, and filtration efficiency—are expected to be transferable to full-scale applications. Nevertheless, further validation under pilot-plant or full-scale operating conditions is recommended to assess the quantitative impact of scale-up fully and to confirm the applicability of the proposed washout weir height modification under real operating conditions.

The filtration media consisted of a dual-layer configuration of sand and activated carbon. The depth ratio and grain sizes were selected based on previous experimental findings from our Master’s study (Refs.^17,18^, which confirmed that this configuration provides stable hydraulic flow, effective solids removal, and minimal head loss. Sand serves as the primary filtration medium, while the activated carbon layer enhances the removal of fine suspended solids and adsorbable compounds. This arrangement also ensures effective media cleaning during washout cycles, maintaining consistent filter performance.

The first run (R1) without modification of the weir height was operated. The results of influent water were illustrated in Table 2, and the results of filtered water of eight consecutive operating days in R1 are summarized in Table 3. Table 4 presents the measurements of washout water for this run.

**Table 2:** Run (R1) input measurements.

Time	Input					
	Av. Q red	Standard deviation (SD)	Av. turbidity	TSS	Temp.	PH
	(L/min)		(NTU)	(mg/l)	(°C)	
Day1	19.60	± 0.09	16.15	250.00	24.50	8.00
Day2	19.55	± 0.09	16.10	250.00	24.50	7.90
Day3	19.60	± 0.09	16.15	250.00	24.50	8.00
Day4	19.61	± 0.10	16.16	250.00	24.00	8.00
Day5	19.63	± 0.08	16.18	250.00	24.50	7.80
Day6	19.60	± 0.05	16.15	250.00	24.50	8.00
Day7	19.55	± 0.09	16.10	250.00	24.00	8.00
Day8	19.65	± 0.10	16.20	250.00	24.50	8.00
ave	19.60	± 0.08	16.15	250.00	24.30	8.00

**Table 3:** Run (R1) output measurements.

Time	Output							TSS
	Av. Q red	Standard deviation (SD)	Av. turbidity	TSS	Standard deviation (SD)	Temp.	PH	R. E
	(L/min)		(NTU)	mg/l		(°C)		%
Day1	18.81	± 0.08	0.76	10.42	± 0.07	25.00	7.70	95.83
Day2	18.76	± 0.08	0.75	10.38	± 0.07	25.00	7.60	95.84
Day3	18.81	± 0.08	0.76	10.42	± 0.07	25.00	7.70	95.83
Day4	18.83	± 0.10	0.76	10.43	± 0.08	24.50	7.70	95.82
Day5	18.84	± 0.07	0.76	10.44	± 0.06	25.00	7.50	95.82
Day6	18.81	± 0.05	0.76	10.42	± 0.04	25.00	7.70	95.83
Day7	18.76	± 0.06	0.75	10.38	± 0.07	24.50	7.70	95.84
Day8	18.86	± 0.10	0.76	10.46	± 0.08	25.00	7.70	95.81
Av.	18.81	± 0.06	0.76	10.42	± 0.06	24.80	7.70	95.83

**Table 4:** Run (R1) washout water measurements.

Time	WASHOUT					Av. H	WSR
	Av. Q red	Av. turbidity	TSS	Temp.	PH	(mm)	
	(L/min)	(NTU)	mg/l	(°C)			
Day1	0.79	383.90	5928.40	25.00	12.50	19.00	95.98
Day2	0.78	382.70	5929.30	25.00	12.30	19.00	95.98
Day3	0.79	383.90	5928.40	25.00	12.40	19.00	95.98
Day4	0.79	384.30	5928.10	24.50	12.50	19.00	95.98
Day5	0.79	384.70	5927.80	25.00	12.20	19.00	95.98
Day6	0.79	383.90	5928.40	25.00	12.40	19.00	95.98
Day7	0.78	382.70	5929.30	24.50	12.40	19.00	95.98
Day8	0.79	385.10	5927.50	25.00	12.50	19.00	95.98
ave	0.79	383.90	5928.40	24.80	12.40	19.00	95.98

Effluent analysis revealed a significant improvement in water quality, with turbidity reduced to approximately 0.76 NTU and total suspended solids (TSS) decreasing to about 10.42 mg/L, yielding an average removal efficiency of 95.83%. This demonstrates the Dyna-Sand filter’s high performance under steady-state operation, with stable hydraulic and chemical conditions throughout the experimental runs. The washout discharge had an average flow rate of 0.788 L/min, with turbidity at 383.9 NTU and TSS concentration of 5928 mg/L. These figures indicate effective removal of accumulated particles during backwashing, confirming that the system prevented clogging and maintained media permeability, achieving a water-saving ratio of 95.98% Water used for washing accounted for nearly 4.17% of the total influent, highlighting the filter’s efficiency.

In the modified configuration (Run R2), raising the effluent weir height by 40 mm improved hydraulic balance and reduced washout water consumption. Tables 5, 6 and 7 summarize the results, which clearly show that optimizing the weir height has improved both water conservation and filtration stability, all without compromising effluent quality.

**Table 5:** Run (R2) input measurements.

Time	Input					
	Av. Q red	Standard deviation (SD)	Av. turbidity	TSS	Temp.	PH
	(L/min)		(NTU)	(mg/l)	(°C)	
Day1	19.81	± 0.15	16.36	250.00	25.00	7.90
Day2	19.66	± 0.10	16.21	250.00	25.50	8.20
Day3	19.63	± 0.08	16.18	250.00	25.00	8.20
Day4	19.61	± 0.08	16.16	250.00	25.50	8.00
Day5	19.65	± 0.05	16.20	250.00	25.50	8.10
Day6	19.60	± 0.09	16.15	250.00	25.50	8.30
Day7	19.60	± 0.09	16.15	250.00	25.00	8.40
Day8	19.60	± 0.09	16.15	250.00	25.50	8.30
ave	19.64	± 0.09	16.19	250.00	25.30	8.20

**Table 6:** Run (R2) output measurements.

Time	Output							TSS
	(L/min)	Standard deviation (SD)	Av. turbidity	TSS	Standard deviation (SD)	Temp.	PH	R.E
	Av. Q red		(NTU)	mg/l		(°C)		%
Day1	19.33	± 0.15	0.77	8.90	± 0.10	25.50	6.90	96.43
Day2	19.18	± 0.10	0.76	8.80	± 0.07	25.50	7.10	96.47
Day3	19.15	± 0.07	0.75	8.79	± 0.05	25.50	7.20	96.48
Day4	19.13	± 0.07	0.75	8.78	± 0.05	26.00	6.90	96.48
Day5	19.16	± 0.05	0.76	8.80	± 0.03	26.00	7.10	96.47
Day6	19.12	± 0.08	0.75	8.78	± 0.06	26.00	7.30	96.48
Day7	19.12	± 0.08	0.75	8.78	± 0.06	25.50	7.40	96.48
Day8	19.12	± 0.08	0.75	8.78	± 0.06	25.50	7.30	96.48
Ave	19.16	± 0.09	0.76	8.80	± 0.06	25.80	7.15	96.47

**Table 7:** Run (R2) washout water measurements.

Time	WASHOUT					Av. H	WSR
	Av. Q red	Av. turbidity	TSS	Temp.	PH	(mm)	
	(L/min)	(NTU)	mg/l	(°c)			
Day1	0.49	631.60	9683.50	25.50	12.30	15.00	97.52
Day2	0.49	625.80	9687.40	25.50	12.80	15.00	97.52
Day3	0.48	624.60	9688.20	25.50	12.90	15.00	97.52
Day4	0.48	624.00	9688.70	26.00	12.50	15.00	97.52
Day5	0.48	625.30	9687.80	26.00	12.70	15.00	97.52
Day6	0.48	623.30	9689.00	26.00	13.10	15.00	97.52
Day7	0.48	623.30	9689.00	25.50	13.20	15.00	97.52
Day8	0.48	623.30	9689.00	25.50	13.10	15.00	97.52
Av.	0.49	625.20	9687.87	25.80	12.83	15.00	97.52

Tables 3 and 4 summarize the results of input raw water and filtered water during the eight-day operation of run (R2). The influent water quality was almost stable, with turbidity ~ 16.2 NTU and TSS fixed at 250 mg/L. The effluent turbidity was reduced to an average of 0.76 NTU, while effluent TSS averaged 8.80 mg/L, yielding a removal efficiency of about 96.47%.

The improvement in TSS removal efficiency (~ 0.67%) was statistically significant based on the experimental uncertainty of the pilot-scale measurements. While the numerical increase is relatively small, it complements the notable reduction in washout water consumption and enhanced hydraulic recovery, highlighting that the combined operational benefits of washout weir modification are practically relevant for continuous sand filtration systems.

The recovery Percentage that simulates the ratio between influent and effluent flows is, on average, 97.52%.

Higher TSS concentrations in the washout water enhance sedimentation efficiency, promoting particle settling and improving water recovery, while minimizing the solid load to downstream sludge handling units. This demonstrates that washout TSS positively contributes to both sedimentation performance and overall system recovery.

Temperature and pH values showed minor fluctuations but remained within a stable operating range. The observed increase in pH in the washout stream can be attributed to the release of accumulated alkaline materials and adsorbed ions from the filter media during the washing process. Table 5 presents the washout water results obtained during the run period. The average washout discharge was in average 0.486 L/min, while washout turbidity increased significantly to about 625 NTU, and TSS rose to an average of 9688 mg/L.

These results demonstrate that the rise of the washout weir achieved an increase in the filtered water amount by about 0.302/min, and so, the unit recovery percentage increased by 1.5%.

The wastewater amount decreased by 0.302/min with almost ratio of about 39% decrease which decreased the washout water percent to ~ 2.5% from the inlet flow.

Also, the rise of the washout weir increased the removal of TSS from water by an average of about ~ 2.5%, which minimized the TSS in the filtered water by 22% for the same loads, hydraulics, and solids.

Compared between before and after raising the weir, the second run results achieved an improvement in the Dynasand productivity by 2.5% and decreased the wastewater amount by 39%. This may be due to the decrease in the water layer above the weir, which also helped in increasing the solids removal and minimizing the solids escape from the weir passage.

The amount of water used in the washout weir ranges between 2.48% of the amount of water entering the dynamic sand filter, with a Water saving ratio of 97.52%. This happened even though the influent loads are very high in solids loads and hydraulic loads, which means the system can stand stand-alone against any solids or hydraulic loads.

The influent water quality remained stable across two runs, with turbidity averaging ~ 16 NTU and TSS maintained at 250 mg/L, confirming uniform experimental conditions. In contrast, effluent quality demonstrated progressive improvement with rising washout weir height. At the start case without modification, effluent turbidity was ~ 0.76 NTU and TSS ~ 10.4 mg/L, corresponding to a TSS removal efficiency of ~ 95.83%. But the modified case reduced effluent TSS to ~ 8.81 mg/L and improved TSS removal efficiency to ~ 96.48%. These results highlight a consistent trend of improved solids removal with higher wash head, indicating that increased wash out weir head enhances filter cleaning and stability, thereby improving effluent quality.

The decrease in pH at the filter outlet compared to the influent can be attributed to the adsorption of basic ions and the accumulation of acidic species within the filter bed. During filtration, carbon dioxide dissolution and biological or chemical oxidation processes may also produce weak acids, slightly lowering the pH of the treated effluent. In contrast, the pH increase observed in the washout water (washout discharge) is mainly due to the release of trapped alkaline materials and accumulated solids from the filter bed. When the washing process is initiated, these basic compounds and fine particles are flushed out, resulting in a temporary rise in pH in the washout stream.

The observed increase in temperature during the experimental runs may be attributed to several factors related to the operating and environmental conditions of the pilot unit. Continuous operation of the filtration system likely caused a gradual rise in temperature due to hydraulic friction between the flowing water and the filter media.

In addition, the pumping system that supplied the influent water might have contributed to heating through mechanical energy conversion. The ambient temperature of the laboratory could also have influenced the water temperature, especially under prolonged testing conditions without active cooling. Furthermore, limited heat dissipation within the closed pilot setup may have allowed thermal accumulation. In some cases, minor biological activity or air–water interaction during the backwash process might have also generated slight thermal variations. Overall, the temperature increase observed throughout the runs is considered a natural consequence of operational and environmental influences rather than a system malfunction.

**Fig. 5:**
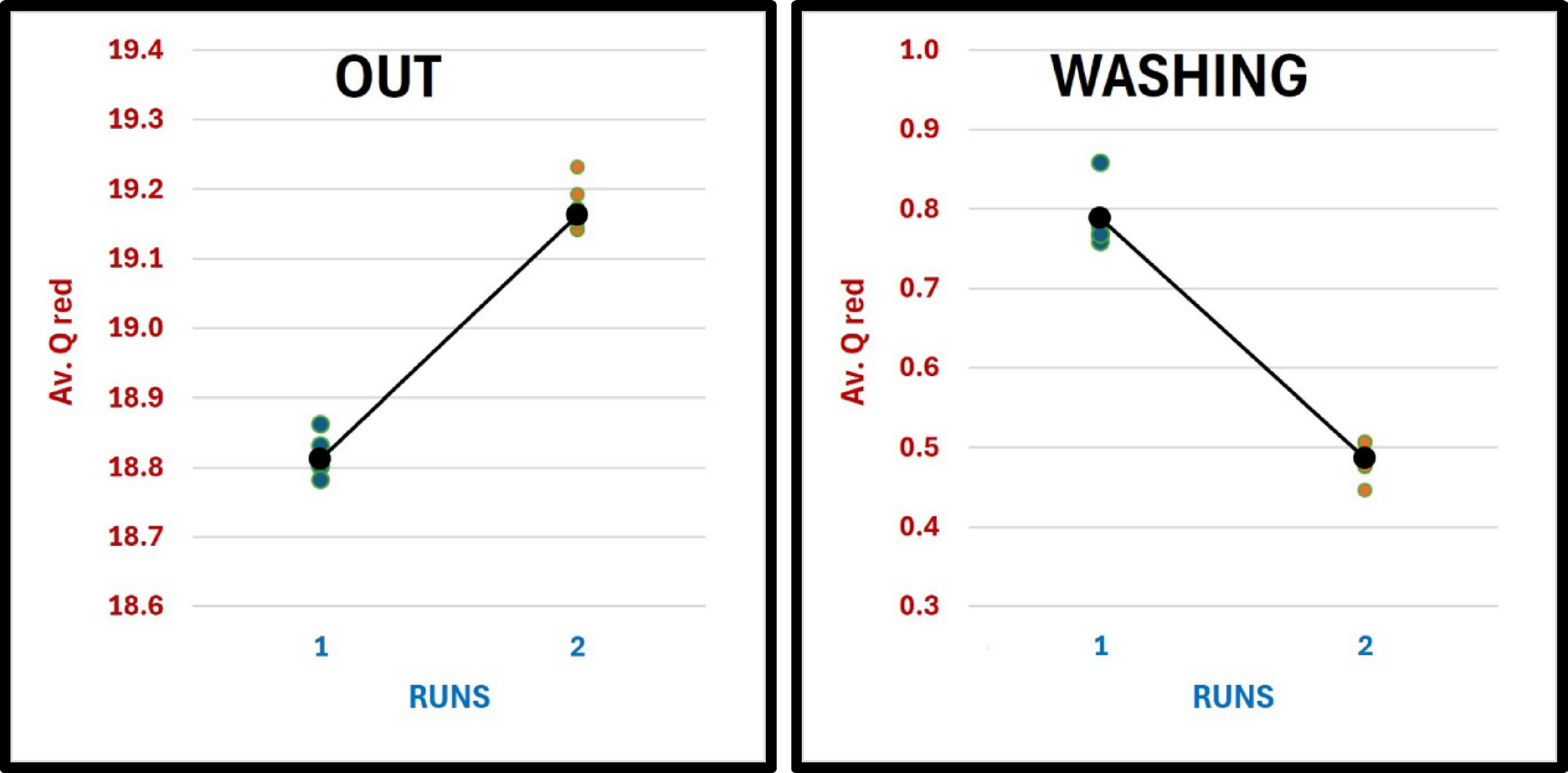
Qin & Qout & Qwash.

Figure 5 illustrates the average flow rates during the two experimental runs. For the first run (R1, H = 0), the influent flow rate (Qin) was 19.60 L/min, the effluent flow rate (Qout) was 18.42 L/min, and the washout water flow rate (Qwash) was 0.79 L/min. In the modified run (R2, H = 40), the influent rate slightly increased to 19.65 L/min, while the effluent reached 18.76 L/min, and the washout water decreased to 0.49 L/min.

This study has some limitations that should be acknowledged. First, the dynamic sand filter was tested using a small-scale laboratory setup, which may not fully represent actual field-scale performance. In addition, the reliance on manual operation and maintenance may have introduced performance variability and measurement uncertainties, affecting the consistency of the results. Furthermore, although the proposed modification successfully reduced the frequency of washout events, the TSS removal efficiency remained nearly unchanged under the hydraulic conditions applied in this study.

## Conclusions

Comparing Run (R1) and Run (R2) shows that raised the washout weir by 4 cm clearly enhanced both the quantity and quality of filtered water. The average removal efficiency improved from 95.83 to 96.47%, representing a 0.67% increase in TSS removal. The effluent TSS concentration decreased from 10.42 to 8.80 mg/L, representing a 15.5% improvement in effluent quality.

The average washout discharge fell from 0.788 to 0.486 L/min, a 38.3% reduction in washout water amount. As a result, filtered water production rose by nearly 2.2%, indicating more efficient hydraulic operation.

These findings confirm that raised the washout weir by 4 cm improved filtration and reduced washout water loss. This, in turn, enhanced system productivity and the quality of washwater produced, assuming a consistent flow rate and solid load.

This study investigated the performance of a dynamic sand filter and evaluated the impact of modifying the washout weir height on filter operation and stability. The experimental results demonstrated that the proposed modification effectively reduced the frequency of washout events and contributed to improved operational stability of the filter. However, under the hydraulic conditions applied in this study, the total suspended solids (TSS) removal efficiency remained nearly unchanged.

Despite the limitations associated with the small-scale laboratory setup and the reliance on manual operation, the findings provide valuable insights into the operational behavior of dynamic sand filters. The results highlight the importance of washout weir height as a key operational parameter influencing filter stability and productivity.

Future research should focus on validating the proposed modification under pilot- and full-scale operating conditions to better represent real field applications. Further investigations are also recommended to assess the effect of varying filtration rates, influent TSS concentrations, and automated operation systems on filter performance. In addition, alternative design and operational modifications should be explored to enhance TSS removal efficiency while maintaining stable and efficient filter operation.

## Data Availability

The datasets used and/or analysed during the current study available from the corresponding author on reasonable request.
