# Correction: Feasibility of modifying the washout water weir on dyna sand filters performance

**DOI:** 10.1038/s41598-026-48241-8

**Published:** 2026-04-10

**Authors:** Esraa Mahmoud Ahmed El Taher, Mohamed El Hosseiny El Nadi, Mohamed Nouh Ahmed Meshref, Amira Mohamed Nagy

**Affiliations:** Faculty of Engineering, ASU, Cairo, Egypt

Correction to: *Scientific Reports* 10.1038/s41598-026-41574-4, published online 24 March 2026

The original version of this Article contained an error in the spelling of the author Esraa Mahmoud Ahmed El Taher which was incorrectly given as Esraa E. M. El Taher.

The original Article has been corrected.

